# Using nurses to identify HAART eligible patients in the Republic of Mozambique: results of a time series analysis

**DOI:** 10.1186/1478-4491-5-7

**Published:** 2007-02-28

**Authors:** Sarah O Gimbel-Sherr, Mark A Micek, Kenneth H Gimbel-Sherr, Thomas Koepsell, James P Hughes, Katherine K Thomas, James Pfeiffer, Stephen S Gloyd

**Affiliations:** 1 Department of Epidemiology, Box 357236, School of Public Health and Community Medicine, University of Washington. Seattle, WA 98195, USA; 2 Health Alliance International, 1107 NE 45th St, Ste 427, Seattle, WA 98105, USA; 3 Department of Health Services, Box 357660, School of Public Health and Community Medicine, University of Washington, Seattle, WA 98195, USA; 4 Department of Biostatistics, Box 357232, University of Washington, Seattle, WA 98195, USA

## Abstract

**Background:**

The most pressing challenge to achieving universal access to highly active anti-retroviral therapy (HAART) in sub-Saharan Africa is the shortage of trained personnel to handle the increased service requirements of rapid roll-out. Overcoming the human resource challenge requires developing innovative models of care provision that improve efficiency of service delivery and rationalize use of limited resources.

**Methods:**

We conducted a time-series intervention trial in two HIV clinics in central Mozambique to discern whether expanding the role of basic-level nurses to stage HIV-positive patients using CD4 counts and WHO-defined criteria would lead to more rapid information on patient status (including identification of HAART eligible patients), increased efficiency in the use of higher-level clinical staff, and increased capacity to start HAART-eligible patients on treatment.

**Results:**

Overall, 1,880 of the HAART-eligible patients were considered in the study of whom 48.5% started HAART, with a median time of 71 days from their initial blood draw. After adjusting for time, expanding the role of nurses to stage patients was associated with more rational use of higher-level clinical staff at one site (Beira OR 1.9, 95% CI 1.1–3.3; Chimoio OR 0.2, 95% CI 0.1–0.5). In multivariate analyses, the rate of starting HAART in patients with CD4 counts of less than 200/mm^3 ^increased over time (HR = 1.07, 95% CI 1.02–1.13), as did the total number of new patients initiating HAART (β = 7.3, 95% CI 1.3–13.3). However, the intervention was not independently associated with either of these outcomes in multivariate analyses (HR = 0.9, 95% CI 0.7–1.2) for starting HAART in patients with CD4 counts of less than 200/mm^3^; (β = -5.2, p = 0.75) for the total number of new patients initiating HAART per month. No effect of the intervention was found in these outcomes when stratifying by site.

**Conclusion:**

The CD4 nurse intervention, when implemented correctly, was associated with a more rational use of higher-level clinical providers, which may improve overall clinic flow and efficient use of the limited supply of human resources. However, this intervention did not lead to an increase in the number of patients starting HAART or a reduction in the time to HAART initiation. Study month appears to play an important role in all outcomes, suggesting that general improvements in clinic efficiency may have overshadowed the effect of the intervention. The lack of observed effect in these outcomes may be due to additional health systems bottlenecks that delay the initiation of treatment in HAART-eligible patients.

## Background

Since 2002 there has been a clear international commitment to expanding the availability of highly active antiretroviral therapy (HAART) in developing countries. The increased political and financial support have resulted in dramatic increases in the number of people in resource-poor countries initiating HAART, reaching over 1.3 million [1] by the end of 2005. Though the results of this expansion have been significant, as of June 2006, only 23% of HAART eligible [2] patients in Sub-Saharan Africa were taking antiretroviral (ARV) treatment.

HIV Care and treatment access is often limited in resource-constrained countries because the health systems in the process of scaling-up HAART are weak, and only recently became oriented toward providing care for chronic diseases. A lack of human resource capacity to handle the increased service requirements of roll-out plans is an important limitation of the public health sector [3-5]. Logistical and operational challenges exist which impede poorer countries' abilities to train, absorb and retain adequate numbers of health workers in public health systems. Political factors, such as macro-level fiscal policies, which restrict hiring of public sector workers, have contributed to the deterioration of human resource capacity and inhibited the rapid scale-up of HIV care and treatment [6,7]. New models of care provision are needed to improve efficiency of existing health services that provide HIV care and treatment including those which rationalize the use of the limited higher level providers through the maximum delegation of tasks within the formal health care team.

In 2004, Mozambique joined a growing number of resource-limited countries heavily affected by HIV/AIDS that began scaling-up national, public sector HIV care and treatment. As of January 2006, national targets were within reach, with over 67,779 individuals enrolled in the HIV-care system nationally, and over 20,805 individuals on HAART [8]. However, in Mozambique, as in the rest of sub-Saharan Africa, there is a severe shortage of qualified clinical personnel, particularly physicians. In 2006, the WHO carried out a global comparison, and classified Mozambique as one of a select group of countries facing a critical shortage of human resources for health, with a density of just 3 physicians and 21 nurses per 100,000 population [9]. 2005 Projections estimated that four-times the current number of doctors would be needed to scale up HAART for all clinically eligible patients within ten years in Mozambique [10]. This projection is only for HAART and does not take into consideration the other pressing needs of the country. In 2006, the two medical schools in the country were far from meeting this demand with only 52 new doctors graduating [11]. This shortage of clinicians leads to severe system inefficiencies and bottlenecks that can delay HAART scale-up. Although training of new personnel has been ongoing and a firm priority of the government of Mozambique, the identification and testing of innovative and flexible strategies to best utilize existing health workers will be necessary to meet the ambitious treatment targets set forth in the national HIV/AIDS strategic plan. In addition, these human resource strategies may provide useful lessons learned for other developing countries with similar human resource and patient flow challenges.

Previous to this study, all diagnostic and curative care within the specialized HIV clinics was provided by physicians or medical officers (for the purposes of this study, the term 'MD/MO' includes both medical doctors and medical officers). Nurses performed baseline clinical assessments and ordered CD4 and other routine laboratory tests in compliance with the core competency guidelines developed for HIV service delivery by the WHO; however, they were not authorized to stage patients using WHO staging criteria or interpret CD4 lab results [12] (a CD4 count measures how strong a person's immune system is, how far HIV disease has advanced and helps predict the risk of complications and debilitating infections). As a result, a large proportion of MD/MO visits occurred with non-HAART eligible patients which decreased MD/MO appointment availability for the sickest patients, thereby reducing the capacity to start new patients on HAART.

**Figure 1:**
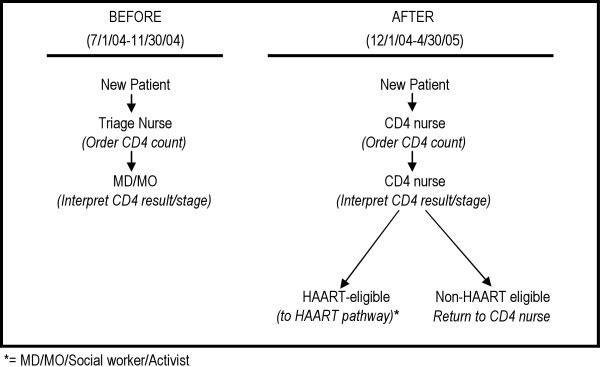
Patient flow before and after intervention.

It was hypothesized that expanding the role of nurses to stage patients using WHO staging criteria and interpret CD4 lab results would lead to more rapid information on patient status, more prompt identification of HAART-eligible patients, fewer losses to follow-up, and ultimately more rational use of limited staff time with higher-level clinical providers (Figure 1). The aim of this study was to discern whether the CD4 nurse intervention increased MD/MO appointment availability and improved the rate at which HAART-eligible patients start ARV treatment.

## Methods

We conducted a time-series intervention trial in two HIV clinics in central Mozambique. These two outpatient HIV clinics were located within two large urban referral hospitals in Beira and Chimoio, the capitals of Sofala and Manica provinces, respectively. These cities lie on the main transport corridor linking the port city of Beira with the Republic of Zimbabwe, and are among the most highly affected areas in terms of HIV prevalence in the country, with the 2004 adult HIV prevalence estimated to exceed 30% in Beira and 25% in Chimoio [13]. Together, these clinics averaged 1600 patient visits per month, of which 1300 were clinical visits and 300 were psychosocial support visits. These HIV clinics were managed and staffed by the Mozambican Ministry of Health (MOH), and received technical and financial assistance from Health Alliance International (HAI), an international NGO with over 18 years of experience providing support to the Mozambican MOH. In June 2004, both sites began receiving MOH-procured antiretroviral (ARV) medicines that were provided free to all HAART-eligible patients.

Staffing at both sites included physicians, medical officers, nurses, social workers, pharmacists and HIV-positive activists. Throughout the study period clinical staff met weekly to discuss patient cases and care coordination. In addition, a HAART eligibility committee, consisting of the HIV clinic manager, MD/MOs, a social worker and a pharmacist, met regularly (varying from daily to every 2 weeks) to confirm HAART eligibility (using clinical and psychosocial readiness guidelines) and to approve initiation of therapy when appropriate.

The CD4 nurse intervention was defined as changing the scope of work for nurses so that they were trained and authorized to evaluate patients' eligibility for HAART using CD4 counts and WHO staging criteria. All nurses participating in the intervention were basic-level nurses with two-years of initial training, and all had attended standardized training on staging HIV-positive patients using CD4 counts and WHO criteria. Prior to the intervention, all HAART and non-HAART eligible patients went through a triage nurse for baseline assessment and blood draws for CD4 counts and were then sent to a MD/MO for their CD4 results, clinical staging, and definition of their next care and treatment steps. After the intervention, all new patients went to the CD4 nurse for initial baseline assessments and blood draws, and then returned to the CD4 nurse to receive their results and undergo clinical staging. Based on the evaluations made by the CD4 nurse, patients were classified as HAART eligible or non-HAART eligible. Depending on their classification, they were then either sent on the 'HAART pathway' (which included referral for appointments with MD/MOs, social workers and other treatment support staff) or scheduled to return to the CD4 nurse for periodic monitoring until deemed HAART-eligible based on clinical and/or laboratory parameters.

**Figure 2:**
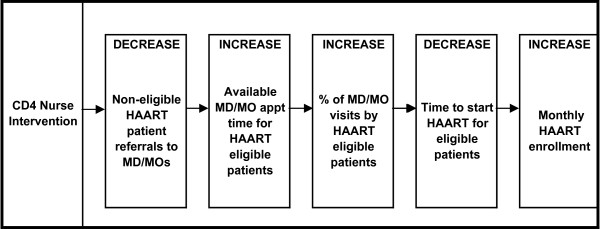
Theoretical model of CD4 nurse intervention.

The theoretical model of this intervention (Figure 2) posited that the increased role of nurses would decrease the number of non-HAART eligible patient referrals to MD/MOs. This decrease in referrals would result in an increase in the availability of MD/MO appointment time for HAART eligible patients, which would increase the proportion of MD/MO visits with HAART eligible patients (outcome 1). It was hypothesized that increasing MD/MO availability for HAART eligible patients, who otherwise may have delayed access to the MD/MO, would allow these sicker patients to move more quickly through the HAART pathway to treatment initiation. Consequently, we expected a decrease in time to start HAART for eligible patients (outcome 2) and an increase in monthly HAART enrollment (outcome 3).

Both sites formally introduced the 'CD4 nurse' intervention in December, 2004. Patients who enrolled at the HIV clinics, underwent initial CD4 testing, or started HAART during the 10-month period between 1 July 2004 and 30 April 2005 were included in the study, with the first five-month period constituting the 'before' period and the second five-month period constituting the 'after' period. During the 10-month period, inclusion in analysis was defined – depending on the study question – as either enrollment at the HIV clinic, starting HAART, or initial CD4 blood draw.

All study subjects were adults of over 15 years of age. Pediatric patients were excluded from this analysis, as CD4 nurses were not authorized to screen this subset of the population.

Study data were derived from existing databases that are maintained at each clinic site and include data routinely collected as part of patient care. These data include basic socio-demographic, clinical, laboratory and pharmacy information for all patients, including the dates of enrollment into the HIV clinic, the dates of all clinical appointments and CD4 tests and the dates of starting HAART.

### Outcome 1

For the first outcome, the proportion of first visits to MD/MOs made by HAART-eligible patients (those with CD4 counts lower than 200/mm^3^) was used to determine if the intervention had the intended effect of limiting non-HAART eligible patient visits to MD/MOs. To calculate this indicator, we selected all adult patients who had initial visits with an MD/MO within 30 days of enrollment at the HIV clinic, and determined the proportion of these patients who were HAART-eligible at the time of their visit. Only patients newly enrolling in the HIV clinics were included in this analysis. Multivariate logistic regression was used to determine the effect of the intervention after adjusting for study month and site.

We determined the sensitivity and specificity of appropriate referrals by nurses. Patients were categorized by their CD4 counts at their initial visit (CD4 counts below 200/mm^3 ^or CD4 counts above 200/mm^3^) and whether a MD/MO visit was performed less than 30 days after enrollment. The sensitivity of appropriate referral was calculated among those with CD4 counts below 200/mm^3 ^and specificity was calculated among those with CD4 counts that were above 200 or were unknown.

### Outcome 2

For the second outcome, the rate of starting eligible patients on HAART was compared before and after the intervention. All adult patients with CD4 counts below 200/mm^3 ^on their first CD4 test between 1 July 2004 and 30 April 2005 were included in this analysis. Cox proportional hazards regression was then used to compare the hazard rates at which these patients started HAART before and after the intervention, using the date of the initial CD4 blood draw as the starting point for follow-up and following patients through December, 2005.

Bivariate analyses using Cox proportional hazards regression was also used to evaluate the bivariate association between the intervention and the promptness of starting HAART, as well as with other variables (study site and study month) hypothesized to potentially influence the rate of starting HAART. A multivariate Cox proportional hazards model was then created to estimate the independent effect of the CD4 nurse on the hazards of starting HAART after adjustment for study month and site.

### Outcome 3

The final analysis determined whether the introduction of the CD4 nurse increased the monthly number of adult patients starting HAART. The outcome was the number of eligible patients started on HAART each month (count variable), compared before and after the intervention. We first evaluated the bivariate associations between the average number of patients starting HAART per month and the presence/absence of the CD4 nurse intervention using linear regression. We also evaluated the bivariate association between the number of patients starting HAART and the study month and site. We then used multivariate linear regression to determine the relationship between the number starting HAART and the presence or absence of the CD4 nurse after adjustment for both study site and study month. Given the time delay inherent in starting eligible patients on HAART, the intervention's effect on this outcome may not have been immediate.

### Covariates

Covariates were considered based on their theoretical plausibility to affect the outcomes included in our analyses. On this basis four main variables were considered as covariates in adjusted analysis for each of the study questions, including the study site, the study month (a measure of the effect of time), the number of monthly MD/MO visits (a measure of MD/MO capacity), and the number of monthly new enrollees at the clinic (a measure of demand). However, we chose to include study month and study site in the final analyses since the number of monthly MD/MO visits and the number of monthly new enrollees did not improve model precision regarding the effect of the CD4 nurse. Study month was included to control for trends over time during the study period and was defined differently for each of the three analyses. For outcome 1 it was defined as the month of enrollment at the HIV clinic; in outcome 2 it was defined as month of initial CD4 blood draw; and in outcome 3 it was defined as month of HAART initiation. Interactions between the site and the CD4 nurse intervention and study month were also tested for each outcome.

The study was approved by the institutional review boards of the National Health Institute, Maputo, Mozambique and the University of Washington, Seattle, USA. Data were analyzed using SPSS version 13.0 (Chicago, IL) and EpiInfo6 (Centers for Disease Control and Prevention, Atlanta, GA).

## Results

Overall enrollment was generally higher in Beira and increased significantly over the study period (Table 1). MD/MO Staffing increased significantly in Beira while in Chimoio it did not. The number of new adult enrollees and total adult MD/MO consults was higher in Beira than Chimoio, and increased at both sites over time. The number of adult enrollees with initial CD4 counts <200 mm^3 ^increased in both sites during the study period, although as a proportion of total enrollees this increase was only significant in Chimoio (27.6% pre-intervention vs. 34.2% post-intervention, p = 0.002).

**Table 1:** Site characteristics

	**Site**	**Pre-intervention ** **(7/04-11/04)**	**Post-intervention ** **(12/04-4/04)**	**p-value**
Mean staffing-adult MD/MO (FTE)	*Beira*	1.9	3.0	0.04**
	*Chimoio*	1.9	2.0	0.42**
Mean no. of new HIV+ adults enrolled per month	*Beira*	272	335	0.03**
	*Chimoio*	172	216	0.14**
Mean no. of adult MD/MO consultations per month	*Beira*	634	681	0.53**
	*Chimoio*	528	686	0.08**
Mean no. of new adult enrollees with initial CD4<200 per month (<30 days after enrollment)*	*Beira*	82	108	0.06**
	*Chimoio*	47	74	0.04**
Proportion of new adult enrollees with initial CD4<200 (as proportion of all new adult enrollees)	*Beira*	30.3%	32.3%	.24±
	*Chimoio*	27.6%	34.2%	.002±
Mean no. of adult MD/MO consultations with new enrollees per month (<30 days after enrollment)	*Beira*	143	414	.02**
	*Chimoio*	142	860	.33**
Proportion of adult MD/MO consults with new enrollees (as proportion of all MD/MO adult consults)	*Beira*	22.6%	12.2%	<.001±
	*Chimoio*	27.0%	25.1%	.09±

* Only includes those enrollees not previously on HAART

**Based on t-tests to compare means

± Based on X^2 ^test of independence

### Outcome 1 – proportion of MD/MO visits by HAART eligibility

In bivariate analysis, the proportion of MD/MO visits with patients with CD4 counts under 200/mm^3 ^increased significantly between the before and after periods at both sites (Table 2). In multivariate analyses, there were significant interactions between site, intervention, and study month (p ≤ 0.001, site × intervention and site × month when simultaneously in the model) and therefore site-stratified analyses were performed. In multivariate analysis controlling for study month, the proportion of initial MD/MO visits with HAART-eligible patients was significantly higher after the CD4 nurse intervention in Beira (OR 1.9, 95% CI 1.1, 3.3) while in Chimoio the effect was reversed (OR 0.2, 95% CI 0.1–0.5).

**Table 2:** Proportion of first MD/MO visits with patients with CD4 counts <200/mm^3^, stratified by site

**Site**	**Variable**	**Bivariate**			**Multivariate**		
		
		**OR**	**(95% CI)**	**p-value**	**OR**	**(95% CI)**	**p-value**
Beira	CD4 nurse intervention (ref = pre-intervention period)	2.2	1.6, 2.8	< 0.001	1.9	1.1, 3.3	0.03
	Study month*	1.1	1.1, 1.2	< 0.001	1.0	0.9, 1.1	0.55
Chimoio	CD4 nurse intervention (ref = pre-intervention period)	2.2	1.6, 3.0	< 0.001	0.2	0.1, 0.5	< 0.001
	Study month*	1.3	1.2, 1.3	< 0.001	1.6	1.4, 1.8	< 0.001

All Odds Ratios and p-values determined through logistic regression

* Defined as patient's enrollment date in HIV clinic. Entered as a continuous variable ranging from 1–10

At both sites, 1551 new enrollees had initial CD4 counts under 200/mm^3 ^and 866 had visits within the first 30 days after enrollment (sensitivity for appropriate visit = 0.56). Sensitivity decreased at both sites between the pre and post intervention periods (Beira pre/post 0.59 vs. 0.36; Chimoio pre/post 0.77 vs. 0.68). Of 3376 patients at both sites with initial CD4 counts under 200/mm^3 ^or unknown, 2313 had no visit (specificity for appropriate visit = 0.69). Specificity increased at both sites over the study period, but the change was more dramatic in Beira (pre/post 0.62 vs. 0.87) than in Chimoio (pre/post 0.52 vs. 0.61).

### Outcome 2 – rate of starting eligible patients on HAART

Of 1880 patients with initial CD4 counts below 200/mm^3 ^during the study period, 911 (48.5%) started HAART by the end of December 2005 with a median time of 71 days from their initial CD4 test. In bivariate analyses, all predictors were significantly associated with starting HAART (Table 3). However, in multivariate analyses, only study month and study site remained significantly associated with time to starting HAART. There was no significant interaction between the study site and the study month, or between the study site and the introduction of the CD4 nurse intervention (data not shown).

**Table 3:** Time to starting HAART in adults by intervention, time and health service characteristics

**Variable**	**Bivariate**			**Multivariate**		
	
	**HR**	**(95% CI)**	**p-value**	**HR**	**(95% CI)**	**p-value**
CD4 nurse Intervention (reference = before)	1.3	1.1, 1.4	0.001	0.9	0.7, 1.2	0.35
Study Month*	1.05	1.03, 1.08	< 0.001	1.07	1.02, 1.13	0.004
Study site (reference = Beira)	1.2	1.1, 1.4	0.002	1.2	1.1, 1.4	0.002

All Hazard Ratios (HR) and p-values determined through Cox proportional hazards regression

* Defined as date of patient's CD4 blood draw. Entered as a continuous variable from 1–10

### Outcome 3: number of patients starting HAART per month

**Figure 3:**
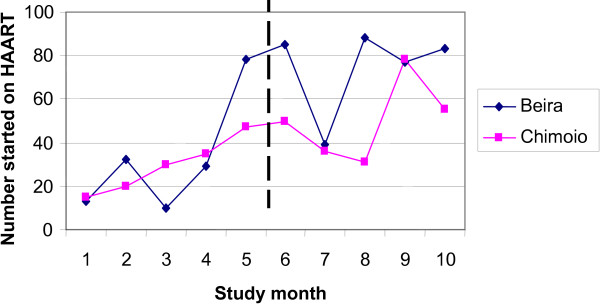
Patients starting HAART by study month.

The number of new patients starting HAART per month increased at both study sites throughout the study period (Figure 3). In bivariate analysis, the number of patients starting HAART was higher after the introduction of the CD4 nurse intervention, but was also significantly associated with study month (Table 4). In multivariate analysis, only study month remained significantly associated with starting HAART. No significant interactions were found between study site and either study month or the introduction of the CD4 nurse intervention (data not shown).

**Table 4:** Associations between number of adults starting HAART and intervention, time, and health service site

**Variable**	Bivariate*			**Multivariate**		
	
	**β**	**(95% CI)**	**p-value**	**β**	**95% CI**	**p-value**
CD4 nurse intervention (reference = pre-intervention)	31.3	11.4, 51.2	0.004	-5.2	-39.4, 29.0	0.75
Study Month**	6.5	3.5, 9.5	< 0.001	7.3	1.3, 13.3	0.02
Study site (reference = Beira)	-13.7	-38.0, 10.6	0.25	-13.7	-30.5, 3.1	0.10

All Odds Ratios and p-values determined through linear regression

* Bivariate analysis adjusted for site

** Defined as date of patient's HAART initiation. Entered as a continuous variable from 1–10

## Conclusion

In this study the CD4 nurse intervention was not positively associated with reduced time to HAART or increased number of adult patients starting HAART per month. However, our findings do suggest that the introduction of the CD4 nurse intervention, when implemented correctly, did increase the proportion of HAART-eligible patients seen by MD/MOs. While not affecting the other outcomes measured, the increased number of MD/MO appointments presumably improves the overall clinic flow and efficiency of these providers particularly where their availability is less. In addition, when sensitivity/specificity analyses were carried out we were able to conclude that the proportion of non-HAART eligible (non-CD4<200/mm^3^) patients that had medical visits lessened over the study period.

In bivariate analysis, there were significant changes seen in the three outcomes of interest. However, after controlling for secular trends in multivariate analysis, the intervention's effectiveness disappeared. Study month appears to play an important role in all outcomes, which suggests that general improvements in clinic and system efficiency were a source of improvements in outcomes over the study period. These time trends may have obscured the potential impact of the CD4 nurse intervention, and the effect of the intervention may have been more apparent had the study occurred later when the system was more stable and enrollment into HAART more consistent.

One important limitation of this study is that external factors, such as health worker trainings, human resource shifts and the influx of AIDS drugs, particularly in the post-intervention period, likely impeded the study's ability to detect a significant impact of the CD4 nurse intervention. For example, in Beira during month seven, there was a large decrease in new patients starting HAART, which we believe was due to factors beyond our control (a social worker training, changes in clinic management during the study period, etc.). The month-to-month random variation in outcomes due to these external factors may have obscured the impact of the intervention. In addition, the data demonstrate that the overall workload is increasing at both sites, as the proportion of visits with new patients reduces and providers are increasingly inundated with 'old' (previous) patients. With increasingly larger numbers of 'old patients' being seen, the rate of starting new patients on HAART (outcome 2) and the monthly mean number of new patients put on HAART (outcome 3) will slow down as the system becomes overburdened. One solution for this inundation will be the eventual opening of new treatment sites in the vicinity which will be able to absorb some of the overflow.

Another important limitation of the study is due to differential implementation of the intervention itself. The results from the first outcome (the proportion of visits with HAART-eligible patients) suggest that the implementation of the intervention may have occurred differently at the two study sites. Discussions with clinic staff after the data analysis was completed revealed that the complete implementation of the intervention was delayed in Chimoio due to the absence of a key clinic advisor. This delay may explain the differences between the effect of the intervention in Chimoio and Beira.

Also, additional steps that occur after patient staging and prior to HAART initiation may independently affect the study outcomes and were not considered for this study. These steps to initiate HAART include 1) attending several sequential visits with social workers and other care providers, 2) passing through adherence building interventions such as mandatory cotrimoxazole prophylaxis regimens, and 3) case review and approval by a multidisciplinary committee designed to improve coordination and quality assurance. Delays at any of these steps may mitigate the positive time gains resulting from the increased MD/MO availability due to the CD4 nurse intervention.

Finally, having the nurse interpret CD4 results and stage patients only reduces visits to the MD/MO for patients with high CD4 counts. For patients with low CD4 counts the new system actually increases the number of patient visits, since the patient must return to the MD/MO and begin the further screening process. Therefore, a greater positive effect may only be seen to the extent that the patient population enrolls earlier on in their disease progression. As HAART continues to roll out in Mozambique and the health system matures, a greater proportion of earlier stage patients may enroll at treatment sites, which may allow for sicker patients to more rapidly receive care and treatment (through a more rapid movement from HIV care enrollment to HAART initiation).

Further research is needed to address these aspects of the study and confirm the effect of the CD4 nurse intervention on clinic functioning and rate of HAART-eligible patients initiating treatment. Future studies of this type should be initiated only after the rate of starting patients on HAART is stable to more clearly differentiate the impact of the intervention from additional factors that may affect the study outcomes. In addition, implementing this study in sites with less MD/MO availability may be more able to demonstrate improved efficiencies related to the intervention. Future research should also endeavor to simultaneously improve training and ongoing supervision for new interventions like the CD4 nurse as well as quantify undefined system bottlenecks that contribute to significant delays in initiating HAART for eligible patients. Although many of these potential bottlenecks are designed to assure quality and coordination of the care team, and improve patient readiness for initiating HAART, they may have the unintended consequence of significantly delaying access to antiretroviral medicines.

## Competing interests

The author(s) declare that they have no competing interests.

## Authors' contributions

SOGS was responsible for the initial conception and design of the data, participated in the data analysis and drafted the original text. MAM participated in the data analysis and made significant comments on progressive drafts. MAM was supported in part through an STD/AIDS Research Training Grant at the time that this study was completed (NIH T32 AI 07140). KHGS participated in the design of the study and made significant comments on progressive drafts. KHGS is a Doris Duke Charitable Foundation (ORACTA) grant recipient. TK provided input on the design of the study and provided comments on progressive drafts of the manuscript. JPH and KKT provided critical input in the data analysis and both made substantive comments on progressive drafts. JP gave input on the development of the discussion section and helped in the revision of final drafts. SSG was instrumental in the initial design of the study question and provided input on subsequent drafts. All authors read and approved the final manuscript.
